# Opsonic Researches in Patients Suffering from Streptococcic Wounds

**Published:** 1919-01

**Authors:** 


### PATHOLOGY AND BACTERIOLOGY

---

***Opsonic Researches in Patients Suffering from Streptococcic Wounds.*** By LE FEVRE de ARRIC. *Comptes rendus de la Société de Biologie,* October 12, 1918

The author tested 63 specimens of serum, 42 belonging to patients suffering from streptococcic wounds, and 21 to control cases, either normal, or presenting no streptococcic lesions.

The method used was Wright's capillary method — the tests were made with both whole and inactivated serum (by heating to 56°). The results were as follows :

(1) The whole sera in streptococcic cases have low opsonic index, inferior to 1.

(2) Inactivated sera of the same cases have high opsonic index, superior to 1.

(3) The whole sera in cases of old streptococcic wounds, tested 6

to 12 weeks after receipt of the wound, sometimes have opsonic index superior to 1, but only rarely.

(4) Inactivated sera in streptococcic cases always have high opsonic powers.

(5) The heating test shows that, except in very few cases, the serum of streptococcic cases is richer in thermostabile opsonins than normal sera.

(6) Generally the opsonic index for one patient increases with the lapse of time after receipt of the wound.

(7) The highest index was found in cases of old wounds, from which the streptococci had disappeared spontaneously.

(8) Some observations show that as the opsonic power of the serum is raised, concomitantly the bacilli prove more easily destroyed by the phagocytes.

(7) Levaditi's cuti-reaction seems to prove that the organism is sensibilized, rather than immunized, for a long time, by the presence of streptococci.

